# Investigation of *Yersinia pestis* Laboratory Adaptation through a Combined Genomics and Proteomics Approach

**DOI:** 10.1371/journal.pone.0142997

**Published:** 2015-11-24

**Authors:** Owen P. Leiser, Eric D. Merkley, Brian H. Clowers, Brooke L. Deatherage Kaiser, Andy Lin, Janine R. Hutchison, Angela M. Melville, David M. Wagner, Paul S. Keim, Jeffrey T. Foster, Helen W. Kreuzer

**Affiliations:** 1 Center for Microbial Genetics and Genomics, Northern Arizona University, Flagstaff, AZ, 86001, United States of America; 2 Chemical and Biological Signature Sciences, Pacific Northwest National Laboratory, Richland, WA, 99352, United States of America; 3 Department of Chemistry, Washington State University, Pullman, WA, 99354, United States of America; University of Mississippi Medical Center, UNITED STATES

## Abstract

The bacterial pathogen *Yersinia pestis*, the cause of plague in humans and animals, normally has a sylvatic lifestyle, cycling between fleas and mammals. In contrast, laboratory-grown *Y*. *pestis* experiences a more constant environment and conditions that it would not normally encounter. The transition from the natural environment to the laboratory results in a vastly different set of selective pressures, and represents what could be considered domestication. Understanding the kinds of adaptations *Y*. *pestis* undergoes as it becomes domesticated will contribute to understanding the basic biology of this important pathogen.

In this study, we performed a parallel serial passage experiment (PSPE) to explore the mechanisms by which *Y*. *pestis* adapts to laboratory conditions, hypothesizing that cells would undergo significant changes in virulence and nutrient acquisition systems. Two wild strains were serially passaged in 12 independent populations each for ~750 generations, after which each population was analyzed using whole-genome sequencing, LC-MS/MS proteomic analysis, and GC/MS metabolomics. We observed considerable parallel evolution in the endpoint populations, detecting multiple independent mutations in *ail*, *pepA*, and *zwf*, suggesting that specific selective pressures are shaping evolutionary responses. Complementary LC-MS/MS proteomic data provide physiological context to the observed mutations, and reveal regulatory changes not necessarily associated with specific mutations, including changes in amino acid metabolism and cell envelope biogenesis. Proteomic data support hypotheses generated by genomic data in addition to suggesting future mechanistic studies, indicating that future whole-genome sequencing studies be designed to leverage proteomics as a critical complement.

## Introduction

Microorganisms inhabiting the natural environment experience vastly different selective pressures from those growing in the laboratory. The natural environment is generally nutrient-limited for microbes, with spatial orientation playing an important role in nutrient acquisition and interaction with neighboring cells [1–3]. In contrast, laboratory conditions are often far removed from those in the environment, with a high likelihood that a cell will find itself in a nutrient-rich environment, growing in a monoculture without interspecies competition, and free from challenges from the host immune system. Little is known about the genomic adaptations arising during the transition from wild to laboratory conditions, largely because whole-genome sequencing has only recently become available as an economically viable means of addressing fine-scale evolutionary questions. Similarly, little is known regarding changes in protein expression arising during pathogen domestication.

*Yersinia pestis* is a recently emerged clone of *Yersinia pseudotuberculosis*, having arisen perhaps as recently as 2,600 years ago [4] and is the causative agent of plague. *Y*. *pestis* has been responsible for three historical pandemics, including the Justinian plague (first pandemic; 6^th^-18^th^ centuries) [5], and the second pandemic from the 14^th^-17^th^ centuries [6]. The third and most recent global plague pandemic has been attributed to a reemergence of *Y*. *pestis* in China in the mid-19^th^ century, with subsequent distribution around the world via commercial shipping vessels [4, 7, 8].

*Y*. *pestis*’ natural life cycle is primarily sylvatic; the organism occurs in specific flea vectors on rodent hosts [7], with humans acting as incidental hosts. It has become endemic in the western United States, with particular foci occurring in the Four Corners region of Arizona, Utah, Colorado, and New Mexico [9]. Endemic plague continues to be an issue for wildlife biologists and human public health worldwide, and a basic understanding of this organism’s biology has important implications for disease control and prevention.

In this study, we explored genomic and proteomic changes arising during laboratory culturing conditions in recently isolated sylvatic strains of *Y*. *pestis*. We performed a parallel serial passage experiment (PSPE), in which multiple populations of two strains were passaged repeatedly under separate but identical growth conditions. This allowed each population to evolve on its own independent trajectory. Although PSPE has previously been carried out on several other *Y*. *pestis* strains [10], the purpose of those studies was to evaluate mutation rates in variable number tandem repeat loci and no whole-genome sequencing was performed. PSPE has been performed extensively with *Escherichia coli* in glucose- and maltose-limiting medium in order to elucidate evolutionary processes (known as the Lenski experiment, it has been running continuously since 1989 and has most recently been described in [11]). This long-term evolution experiment is ongoing, and has selected strongly for mutations that improve cells’ ability to take up and utilize glucose/maltose. The *E*. *coli* experiment imposes a fairly stringent set of evolutionary pressures on a strain pre-adapted to laboratory growth. In our case selective pressure is relaxed due to the rich medium used, and cells are presented with multiple potential evolutionary pathways for adaptation from environmental to laboratory growth.

Mikkola and Kurland [12] have reported isolation and adaptation of wild *E*. *coli* cells, and Sjödin et al [13] have compared wild and laboratory strains of *Francisella tularensis*, but did not directly investigate mechanisms of domestication. Eydallin et al [14] investigated metabolic and phenotypic consequences of *E*. *coli* domestication, but did not explore the underlying genetic causes of observed phenotypes. Although Saxer et al [15] recently used proteomics and genomics to examine adaptation by two commensal bacteria to laboratory growth, to our knowledge this is the first reported experiment investigating specific genomic and proteomic adaptation(s) of a wild pathogen to laboratory conditions. *Y*. *pestis* has been the subject of numerous proteomic studies investigating protein expression changes caused by temperature [16, 17] and iron availability [18] related to the transition between flea and mammalian hosts, and by intracellular growth in macrophages [19]. Proteomic approaches have also been used for the identification of virulence factors and mechanisms [20, 21] and for strain identification [22]. The purpose of this study was to conduct a thorough genomic and proteomic characterization of two wild *Y*. *pestis* strains passaged under laboratory conditions for ~750 generations through whole genome sequencing to discover single nucleotide polymorphisms (SNPs) and insertion/deletion mutations (indels), as well as LC-MS/MS proteomic analysis and GC-MS carbohydrate profiling to examine protein and monosaccharide abundance changes over time. The picture that emerges from the combined proteomic and genomic data is of a systems-level change in metabolism and physiology that results from a complex interplay between genetic and regulatory factors. Our results illustrate the value of systems-level phenotypic measurements in PSPE studies and suggest several promising avenues for future investigations.

## Materials and Methods

### Growth media, chemicals, and growth conditions

Unless otherwise noted, all chemicals and reagents used in this study were of analytical grade. All culturing of *Y*. *pestis* was carried out in a Centers for Disease Control and Prevention-approved Select Agent BSL3 facility at Northern Arizona University. *Y*. *pestis* strains were routinely cultured in Brain-Heart Infusion broth (BHI; BD Diagnostics), and, as required, on 5% sheep’s blood agar (SBA; Hardy Diagnostics) or BHI supplemented with 1.5% w/v agar. Cells were grown at 28°C to minimize the likelihood of plasmid loss, with vigorous agitation under aerobic conditions.

### Isolation of Yersinia pestis strains used in this study

Two wild *Yersinia pestis* strains were isolated from fleas using methods developed in this laboratory [23, 24]. Briefly, fleas were collected from black-tailed prairie dog (*Cynomys ludovicianus*) colonies in Texas, USA in 2009 (Yp1945) and from Gunnison’s prairie dog colonies (*C*. *gunnisoni*) in Arizona, USA in 2011 (Yp2126). Both colonies had shown signs of recent die-offs. The Arizona sample was collected from public land, and a research permit was provided by the State of Arizona for DMW. The Texas sample was collected from private land by a State of Texas veterinarian, who was given permission by the landowner to conduct the study at the site. Because fleas were collected from burrows after rodent hosts had already died, no prairie dogs were harmed or killed for the purpose of this study. Fleas were pooled by burrow and homogenized in BHI broth supplemented with 10% glycerol. The homogenized suspensions were plated onto cefsulodin, irgasan, and novobiocin (CIN) agar plates and incubated at 28°C for 48 h. Suspected *Y*. *pestis* colonies were purified onto SBA, and their identity confirmed by a real-time PCR-based assay targeting the plasmid-borne *pla* gene [25, 26]. Confirmed *Y*. *pestis* isolates were spread onto a fresh sheep blood agar plate. All colonies were scraped from this plate and used to make a freezer stock, which served as the inoculum for the starting culture for PSPE. Using this protocol, the *Y*. *pestis* strains used were passaged no more than three times during the isolation process.

### Parallel Serial Passage Experiment (PSPE)

**Fig 1:**
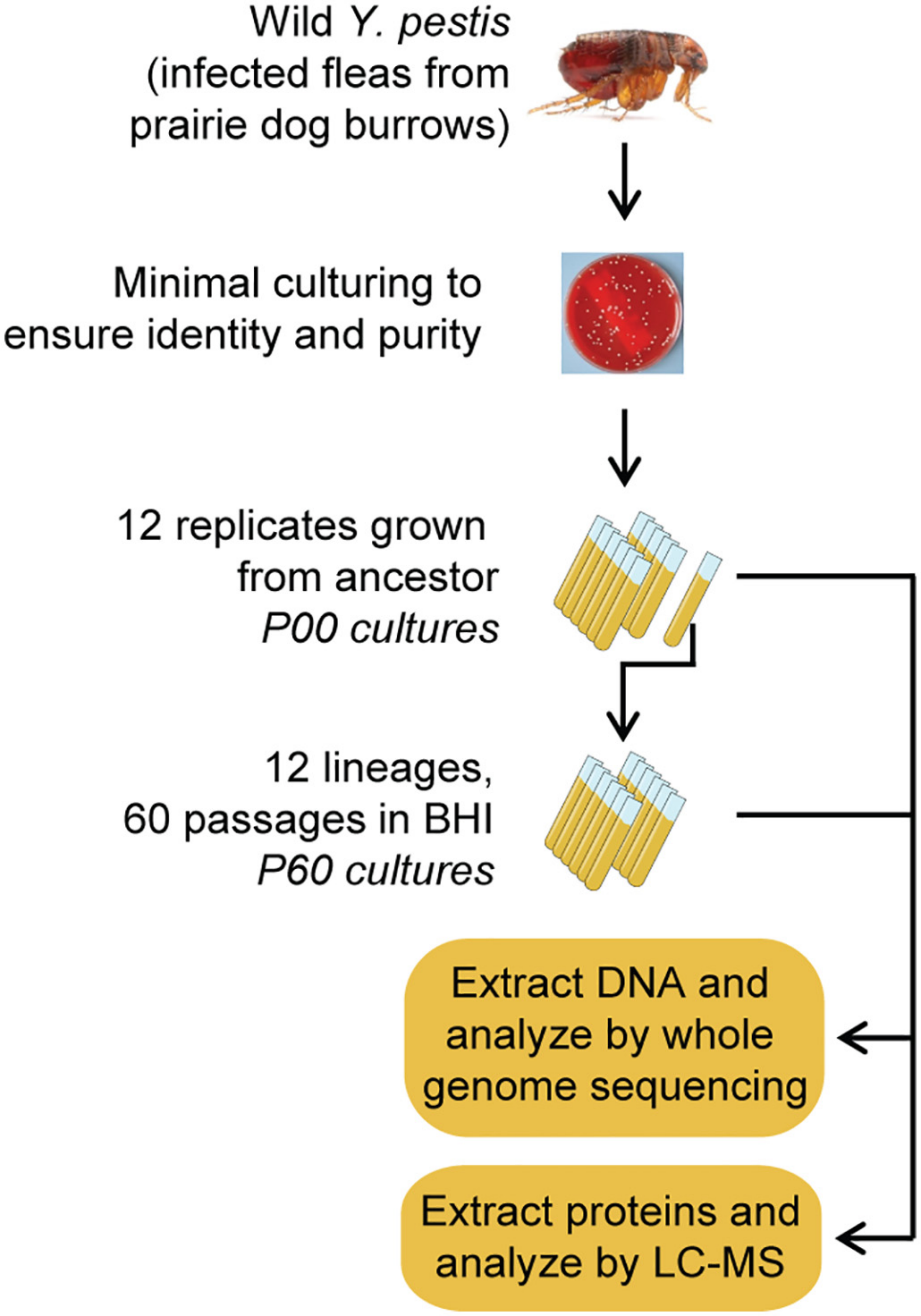
Simplified outline of PSPE. Following isolation and minimal laboratory culturing (≤3 passages on solid media), each isolate was grown in replicate broth cultures. A single culture was used to inoculate replicate 12 independent broth cultures carried through 60 passages (~750 generations). Evolved genomes and proteomes were compared to ancestral states.

The conceptual framework of this experiment is outlined in Fig 1. PSPE was carried out by repeatedly passaging the two *Y*. *pestis* strains in 10 ml BHI broth at 28°C for 48 h (stationary phase) in 50 ml conical tubes. Passages destined for proteomic and carbohydrate analyses at Pacific Northwest National Laboratory (PNNL) were grown in 15 ml BHI in order to achieve sufficient cellular biomass. In order to minimize the number of passages before the start of the experiment, Passage 0 (P00) cultures were inoculated directly from frozen glycerol stocks into two replicate 15 ml BHI broth cultures and agitated vigorously during growth. An additional ten replicate cultures were later inoculated and processed to provide sufficient statistical resolution (see below) for proteomic analysis of starting strains. Beginning with P01, twelve populations were inoculated, originating from one of the original cultures for each strain. Cell density was determined by measuring optical density (600 nm) prior to subculturing to ensure sufficient inoculum was used. To avoid a potential genetic bottleneck resulting in the loss or fixation of mutant genotype(s) by means other than selection, approximately 10^6^ CFU were used as an inoculum for each passage. Passages 1–60 were carried out by diluting each stationary phase culture in two 10-fold dilutions, routinely 100 μl into 900 μl BHI broth, with 250 μl of the final dilution inoculated into 10 ml fresh BHI broth (i.e. 10^1^ * 10^1^ * 4x10^1^ = 4x10^3^). As noted above, populations destined for proteomic analysis in addition to DNA sequencing were grown in 15 ml BHI broth. Under the conditions of this experiment, cell populations reached approximately 5 x 10^8^ CFU ml^-1^ representing ~12 generations per passage in 10 ml cultures and ~17 generations in 15 ml cultures, and resulting in ~750 generations per population over the course of the experiment. Population 1 (L01) of Yp2126 became contaminated early (passage 3) in the experiment; this contaminant was later identified as *Bacillus licheniformis* (a common laboratory contaminant) by 16S sequencing and the culture was discarded, leaving a total of eleven independent populations for Yp2126 and twelve populations for Yp1945.

### DNA extraction and sequencing

Total cellular DNA from endpoint populations and starting isolates was extracted from 250 μl stationary phase culture (approximately 1.5 x 10^8^ cells) using the DNeasy Blood and Tissue Kit (Qiagen, Valencia CA) according to the manufacturer’s instructions. Cells were pelleted by centrifugation at 5,000 x *g* and supernatant removed prior to processing.

DNA extracts were prepared for sequencing according to the manufacturer’s instructions, with the following modifications: DNA was fragmented using a SonicMan 96-well sonicator (Matrical Bioscience, Spokane WA) to yield a distribution of fragment sizes between 250–500 bp. After sonication, fragmented DNA was purified using a QIAquick PCR Purification kit (Qiagen, Valencia CA). All other purification steps were carried out using Agencourt AMPure XP beads (Beckman Coulter, Danvers MA) in a 96-well format. End repair, dA-tailing, and ligation reactions were carried out using NEBNext kit (New England Biolabs, Ipswich MA). Size selection was performed using E-Gel SizeSelect Gels (Life Technologies, Foster City CA). AIR DNA Barcodes– 48 (Bioo Scientific, Austin TX) were used during indexing PCR. Quantification of prepared libraries was carried out using Kapa SYBR Fast ABI Prism 2x qPCR Master Mix (Kapa Biosystems, Boston MA) on an Applied Biosystems ABI Prism 7900 (Life Technologies, Foster City CA). Flow cells were prepared for sequencing on a Cluster Station (Illumina, San Diego CA). Paired-end DNA sequencing was carried out on an Illumina Genome Analyzer IIx or MiSeq (Illumina, San Diego CA) using SBS version 3 reagents at the Translational Genomics Research Institute Division of Pathogen Genomics (TGen North, Flagstaff AZ), or using an Illumina MiSeq at the NAU Environmental Genetics and Genomics Laboratory(from 50 and 200 base reads). Read length varied depending on the library preparation chemistry and sequencer available at the time of preparation. Raw reads from evolved populations in FASTQ format have been deposited in the Sequence Read Archive (SRA; accession numbers SRR2183237-SRR2183258, and SRR2183308).

### Genome assembly and mutation analyses

Ancestral genomes from Yp1945 and Yp2126 were assembled from Illumina sequence reads (FASTQ) using ABySS v1.3.2 [27] in order to uncover large-scale genome rearrangements relative to YpCO92 reference. Because average fold-coverage for the chromosome and plasmids pCD1, pMT1, and pPCP1 was excessive for genome assembly when aligned to *Y*. *pestis* CO92 (accession numbers NC_003143, NC_003131, NC_003134, and NC_003132, respectively), and indeed was likely to generate misassemblies due to the accumulation of sequencing errors, we randomly down sampled to 7,000,000 read pairs for each strain before generating assemblies using CO92 as a reference sequence. Post-assembly correction using the PAGIT toolkit [28] yielded 12 contigs for Yp1945 and 19 contigs for Yp2126. Visualization of assembled contigs relative to YpCO92 reference was performed using MUMmer v3.23 [29]. Both assemblies have been deposited as Whole Genome Shotgun projects in GenBank (accession numbers LIXX00000000 and LIXY00000000)

Single nucleotide polymorphism (SNP), insertions/deletions (indels), and small-scale rearrangement (e.g. transposons) analyses for all strains were carried out using breseq v0.24-rc6 (https://code.google.com/p/breseq/downloads/list) using—j2—p—c arguments and a customized YpCO92 annotated GenBank file as reference, generated by concatenating GenBank files of the chromosome and all three plasmids. Mutations were considered significant if they were present on at least 90% of reads for ancestral strains, or at least 75% of reads for evolved populations, and were verified by manually examining alignments using Tablet v1.14.04.10 (http://bioinf.hutton.ac.uk/tablet). Mutations called in all populations were discarded, as it is highly unlikely to observe the same mutation in all populations and these are most parsimoniously explained as sequencing errors.

### Inactivation of cells with ethanol and preparation of cells for proteomic analysis

Inactivation of *Y*. *pestis* cells prior to shipment was carried out as described in Lin et al [30], which has been demonstrated to be compatible with downstream proteomic analysis. Approximately 10^9^ stationary phase cells were pelleted by centrifugation. A 500 μl aliquot of culture supernatant was filter-sterilized using 0.22 μm centrifugal filters (Corning); the remaining supernatant was discarded. Cells were inactivated by resuspending cell pellets in 40% ethanol and incubating for 20 min at room temperature. Inactivated cells were washed once with 1 ml sterile phosphate-buffered saline (PBS) and resuspended in 500 μl PBS. Supernatant and cell mass were verified sterile prior to shipment on ice to PNNL. For the purposes of statistical analysis, 10 additional cultures of starting isolates were grown and processed in the same manner as the original cultures, bringing the total number of independent cultures to 12.

### Preparation of samples for proteomic analysis

For peptide preparation, 25–50 μl of ethanol-inactivated *Y*. *pestis* cells were pelleted by centrifugation. Volumes varied because the initial blocking and randomization of run order required more analytical replicates of ancestor samples, and thus a higher starting biomass (see below). Pellets were resuspended in 50 μl lysis buffer (6 M urea (Sigma) and 14.3 mM 2-mercaptoethanol (Sigma) in 100 mM triethylammonium bicarbonate (TEAB) pH 9 (Sigma)). Samples were then incubated for one hour at 60°C with shaking. Insoluble cell material was removed by a brief centrifugation. 400 μl of 100 mM TEAB was added to the supernatant followed by 5 μl 375 mM iodacetamide (Pierce) in 100 mM TEAB. Samples were incubated for 30 minutes at room temperature in the dark. Proteins were digested with 2.5 μg of trypsin (Promega) for 14 hours at 37°C with gentle shaking. Solid phase extraction (SPE) was performed with a vacuum manifold using Strata C-18T columns according to the manufacturer’s instructions. Briefly, 1 ml of 100% methanol (Sigma) was added to activate the resin, followed by a conditioning rinse of 0.1% v/v trifluoroacetic acid (TFA, Sigma), then addition of samples in TEAB. Samples were washed with 0.1% v/v TFA, and eluted with 80% v/v acetonitrile (Sigma) in 0.1% v/v TFA into clean low-protein binding 1.5 ml microfuge tubes (Fisher Scientific). Samples were dried to near completeness (~5 μl) using an Eppendorf Vacufuge Plus. Peptides were resuspended in 0.1% v/v formic acid (Suprapure EMD) in water and the concentration was adjusted to 1 mg ml^-1^ using the BCA assay (Pierce). Samples were transferred to high performance liquid chromatography (HPLC) vials with inert glass inserts and capped with screw caps, and stored at -20°C until analysis.

### Liquid chromatography-mass spectrometry measurements

Digested peptide samples were subjected to liquid chromatography on an Agilent Infinity 1260 HPLC system. The column was a fused silica capillary (40 cm x 150 μm inner diameter) packed with 5 μm particle size, 300 Å pore size Jupiter C18 resin (Phenomenex, Torrance CA). 1 μl aliquots (total mass ~1 μg) were injected and subjected to the following 160-minute gradient: 100% Solvent A for 10 minutes; 0%-7.5% Solvent B over 1 minute; 7.5%-45% Solvent B over 109 minutes; 45%-95% Solvent B over 2 minutes; 95% Solvent B for 10 minutes; 95%-0% Solvent B over 4 minutes; 100% Solvent A for 23 minutes. Solvent A was 5% v/v acetonitrile/0.1% v/v formic acid and Solvent B was 95% v/v acetonitrile/0.1% v/v formic acid. Blanks consisting of 5 μl injections of 50% v/v isopropanol/50% v/v acetone/0.1% v/v formic acid were run with a shorter gradient between samples to minimize column carryover.

Each batch of samples, which comprised P00 and six P60 populations, was run in a block with a randomized run order. Although some MS drift was observed the effect was minimal and did not affect data analysis. Each block was repeated a total of three times with a different random run order each time. To monitor the quality of the chromatographic separation, standards were run before and after each block. The standard was a tryptic digest of ovalbumin, bovine serum albumin, bovine αS1-casein, and bovine lactalbumin (all from Sigma) at equal mass concentrations.

The HPLC was coupled to a Thermo Scientific LTQ Orbitrap XL mass spectrometer via a custom electrospray emitter consisting of an etched fused silica capillary [31]. The MS was operated in data dependent “high-low” mode with a high-resolution (*R* = 30,000) precursor scan collected in the Orbitrap followed by collision-induced dissociation (CID) fragment scans of the top seven most intense precursors collected in the ion trap. Data dependent acquisition parameters were: dynamic exclusion repeat count 2, repeat duration 30 seconds, exclusion list size 250, exclusion list duration 180 seconds.

### Proteomic data analysis

We used intensity-based label-free quantitation (LFQ) using MaxQuant v1.5.2.8 [32, 33], which provided detailed and quantitative data in addition to identifying a large number of proteins.

The protein sequence database used was built by annotating the assembled genomes of wild isolates (i.e. Yp1945 and Yp2126) using RAST [34, 35]. The experimentally determined genome sequences of the starting isolates were used in the database searches for the respective ancestor and evolved populations. Gene/locus names were mapped back to the published *Y*. *pestis* CO92 genome [36] using BLASTp [37]. LC-MS/MS data in the Thermo Scientific RAW file format and the protein sequence database in FASTA format were loaded into MaxQuant.

Peptide identification was accomplished in MaxQuant using the integrated Andromeda search engine [38] with the following parameters: precursor ion mass tolerance, 20 ppm for the first search and 4.5 ppm for the main search; tryptic enzyme specificity; maximum number of missed cleavages, 2; methionine oxidation, and protein N-terminal acetylation were included as variable modifications, and cysteine carbamidomethylation as a fixed modification; minimum number of peptides, 1 (additional analysis was performed using 2 peptide minimum; see below). The default 1% false discovery rate filter was used at both the peptide and protein level. The “match between runs” and “re-quantify” options were also used. Proteomic data have been deposited in the ProteomeXchange Consortium [39] via the PRIDE partner repository (accession numbers PXD002955 and PXD002961).

MaxQuant’s LFQ functionality was also used for quantification [40, 41]. Each dataset was compared to at least three and on average six other datasets for estimation of normalization coefficients. Statistical analysis of LFQ intensity values was carried out using Inferno (http://omics.pnl.gov/software/infernordn), a freely available version of DAnTE [42]. Proteins were judged to have changed abundance significantly if the *q*-value from ANOVA (comparing all P00 replicates to all P60 replicates) was less than 0.05, and the fold change between two conditions was greater than 2; or if the protein was detected in only one condition, and in that case more than half the LC-MS/MS analyses for that condition. Identified proteins were functionally classified using eggNOG v4.1beta [43] based on UniProt identification numbers.

### Carbohydrate analysis

Carbohydrate content of the biomass and medium samples was measured as the monosaccharide profile by the alditol acetate method [44–46]. Briefly, biomass samples were hydrolyzed with H_2_SO_4_, and the resulting monosaccharides were purified by SPE on a C18 stationary phase, converted to their volatile alditol acetate derivatives by reduction with sodium borodeuteride, acetylated with acetic anhydride, extracted with chloroform, purified again by reaction with ammonium hydroxide, followed by SPE on a hydrophilic stationary phase. The organic phase was evaporated to dryness and the sample dissolved in the appropriate amount of solvent for gas chromatography-mass spectrometry (GC-MS) analysis.

GC-MS analysis and quantitation of carbohydrates were carried out as described previously [44–46]. The polyamine putrescine (butane-1,4-diamine), although not a carbohydrate, was initially detected in these experiments as a prominent unknown peak. MS/MS and accurate mass data confirmed the identity of this peak, and putrescine was added to the external standard mixture in subsequent experiments to allow quantitation.

## Results

### Ancestral genome assembly and analysis

Assembled genomes of the ancestral strains, Yp1945 and Yp2126 had no apparent large-scale chromosomal rearrangements when compared to YpCO92 using MUMmer (data not shown). Yp1945 was assembled into 12 contigs with average contig length (N50) of 798,621 and total sequence of 4.82 Mb. Yp2126 was assembled to 19 contigs with N50 of 903,789 and total sequence of 4.81 Mb. A total of 42 SNPs in Yp1945 (5 synonymous, 19 nonsynonymous, 17 intergenic, and 1 noncoding RNA; S2 Table) and 40 SNPs in Yp2126 (5 synonymous, 16 nonsynonymous, and 19 intergenic; S3 Table) differentiated the ancestral strains from YpCO92. The high ratio of nonsynonymous to synonymous SNPs may imply positive selection as these two lineages have become established in their respective niches. In addition to SNPs, Yp1945 contained 38 indels relative to YpCO92 (S2 Table). Yp2126 differed from YpCO92 at 30 indels (S3 Table). All of these mutations were removed from SNP/indel calls for the respective evolved populations using the gdtools SUBTRACT function of breseq to remove spurious calls, i.e. mutations that were present in the genome at the beginning of the experiment but which differ from the YpCO92 sequence used as the reference.

### Mutational analysis of populations after laboratory evolution

We found that the loss of the *pgm* locus and the pCD1 plasmid were common, along with mutations in the genes *ail*, *pepA*, and *zwf*. We defined mutations as dominant in a given population if they were observed in >75% of sequence reads, in order to focus our analysis on mutations evidently well on the way to being fixed. A total of 75 mutations were identified across the 23 populations, with nearly half (n = 36) observed in intergenic regions. Mutations occurred in a wide variety of genes (S1 Spreadsheet), with the overwhelming majority of SNPs (24 of 30) resulting in nonsynonymous amino acid changes. Although intergenic mutations could have effects on gene expression, e.g. by affecting promoter or operator regions, any such effects were not investigated in this study. As our objective was to identify common pathways by which wild pathogens adapt to the laboratory, we looked for mutations arising in a given gene in populations derived from both starting strains, and have restricted further analysis here to genes meeting this criterion. Mutations in *ail*, *pepA*, and *zwf* as well as loss of pCD1 were observed in populations derived from both ancestral strains, but loss of *pgm* was only observed in populations derived from Yp1945 (see below).

### Mutations in *ail* give rise to predicted truncated Ail protein

*Ail* (YPO2905) encodes an adhesin necessary for successful *Y*. *pestis* invasion of host cells [47]. It folds into a monomeric β-barrel protein in the outer membrane [48, 49], and is transcribed at a very high level in *Y*. *pestis* [50, 51]. *Ail* mutations were dominant in 10 of 12 Yp1945-derived populations and 6 of 11 Yp2126-derived populations (Table 1). *Ail* was mutated in two additional populations derived from Yp1945, although read frequency (29%, L06; 32%, L11) did not meet our cutoff for inclusion. All of the observed mutations resulted in either premature stop codons, or, in three populations, disruption by mobile elements.

**Table 1:** Effect of *ail* alleles on Ail protein abundance.

Population	Observed mutation	Ail protein abundance ratio^1^
Yp1945-L01	IS*100* insertion^2^ at nt 21	Ancestor only^3^
Yp1945-L02	E29*	0.021^5^
Yp1945-L03	IS*100* insertion at nt 19	0.007^5^
Yp1945-L04	+20 bp^4^ at nt 142; F69*	Ancestor only
Yp1945-L05	WT	0.011^5^
Yp1945-L06	E62* (29% of reads)	0.902±0.587
Yp1945-L07	E29*	0.020±0.013
Yp1945-L08	+8 bp at nt 523; E182*	Ancestor only
Yp1945-L09	+8 bp at nt 523; E182*	Ancestor only
Yp1945-L10	+1 bp at nt 41; K18*	Ancestor only
Yp1945-L11	W67* (32% of reads)	Ancestor only
Yp1945-L12	Q132*	0.050±0.033
Yp2126-L02	WT	Ancestor only
Yp2126-L03	+4 bp at nt 43; F15*	Ancestor only
Yp2126-L04	E62*	Ancestor only
Yp2126-L05	+4 bp at nt 43; F15*	Ancestor only
Yp2126-L06	-34 bp at nt 122–155; T43*	Ancestor only
Yp2126-L07	WT	Ancestor only
Yp2126-L08	+8 bp at nt 531; E182*	Ancestor only
Yp2126-L09	IS*100* insertion at nt 43 (40% of reads) IS*100* insertion at nt 167 (60% of reads)	Ancestor only
Yp2126-L10	WT	Ancestor only
Yp2126-L11	WT	Ancestor only
Yp2126-L12	WT	0.026±0.015

^1^Ratio of protein abundance of evolved population relative to ancestor. Error reflects standard deviation of the mean using triplicate technical replicates.

^2^Insertion sequence mediated mutations are referred to by their nucleotide position (nt, nucleotide).

^3^Ancestor only, protein was observed in ancestor but not in evolved population.

^4^Indel mutations resulting in frame shift and premature stop codon are indicated by the relevant amino acid mutation

* (bp, base pair).

^5^Protein was only identified in one of three technical replicates; therefore statistical analysis of variance was impossible.

Observed protein levels in evolved strains were consistent with disruptive mutations. Ail protein was not identified in most of the evolved populations, either due to complete absence or presence at levels below detectable limits of LC-MS/MS, and when identified was significantly reduced in abundance relative to ancestral strains. The notable exception to this trend is Yp1945-L06, with 29% of the population harboring an E62* mutation but with Ail levels approximately the same as ancestral cells.

### PepA mutations result in downstream changes to CarAB expression

**Fig 2:**
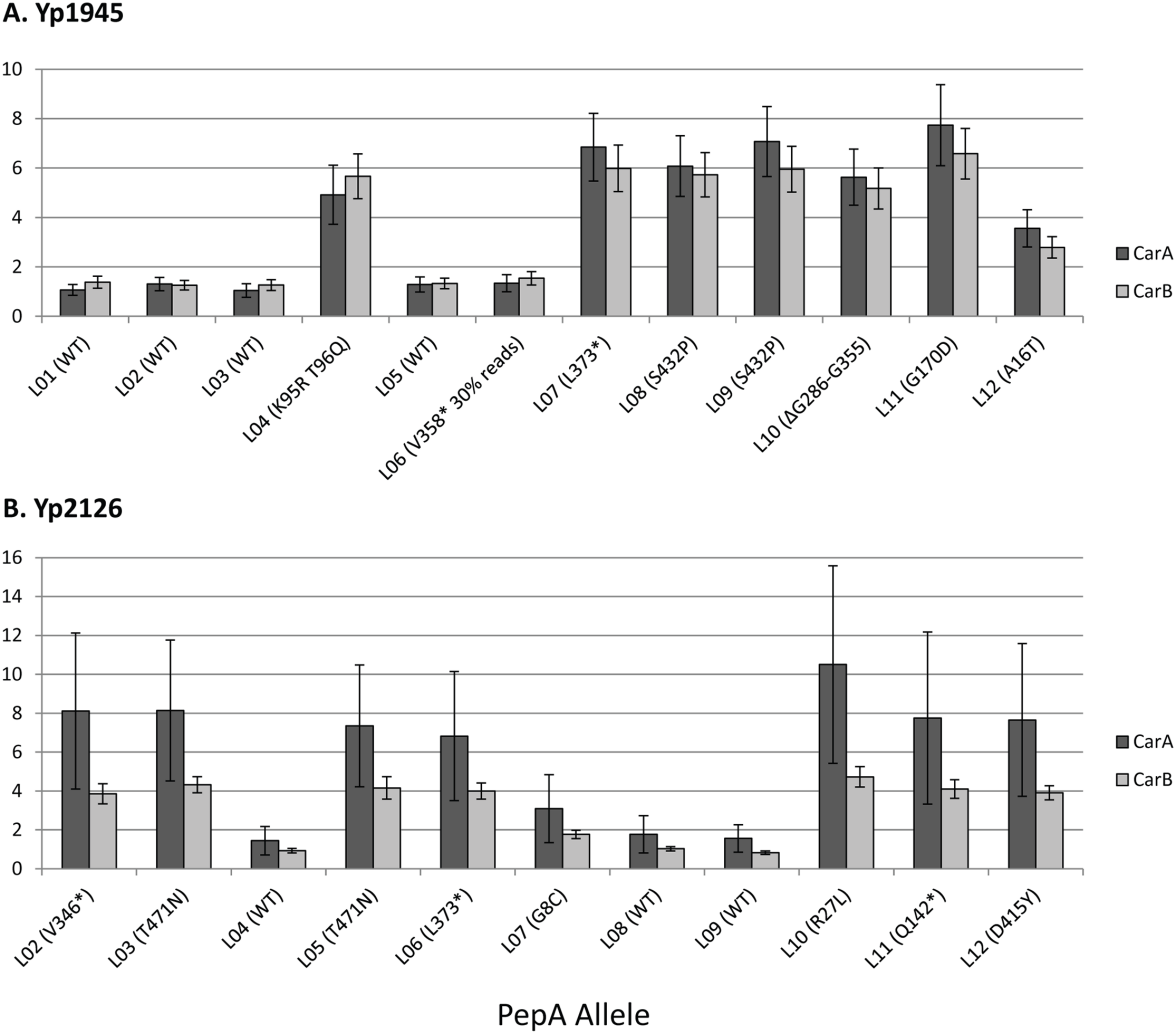
Effects of mutant PepA protein on CarAB protein levels. Evolved populations expressing mutant PepA protein have increased CarA (dark grey bars) and CarB (light grey bars) relative to ancestral cells, consistent with derepression of the *carAB* operon. Each population was measured in triplicate. Specific *pepA* alleles are given in parentheses. Error bars reflect standard deviation of the mean. A, Yp1945-derived populations. B, Yp2126-derived populations.

*PepA* (YPO3441) encodes a multifunctional leucyl aminopeptidase/DNA binding protein [52]. Aside from its eponymous function, it is an accessory protein required for proper plasmid resolution partitioning via the Xer system [53], is required for stable maintenance of P1 prophage [54], and is an accessory protein to Cer recombination [55]. At the end of 60 passages, mutant *pepA* alleles were dominant in seven populations for Yp1945 and eight populations for Yp2126, with an additional population, Yp1945-L06 harboring a mutation at moderate frequency (30% of reads) (Fig 2). When functioning as a DNA binding protein, PepA represses transcription of *carAB*, encoding the large and small subunits of carbamoylphosphate synthetase, by binding to sites upstream of *carA* [52, 56]. CarAB (YPO0481/0482) catalyzes the synthesis of carbamoylphosphate, an early step in arginine and pyrimidine metabolism [57–59]. As PepA represses *carAB* transcription, we hypothesized that the observed mutations serve to de-repress *carAB* transcription.

Proteomic data support this hypothesis. Yp1945-derived populations expressing mutant PepA have on average 6.0-fold higher protein levels of CarA and 5.4-fold higher levels of CarB than their wild progenitors (Fig 2A). Populations derived from Yp2126 expressing mutant PepA have on average 7.4-fold higher protein levels of CarA and 3.9-fold higher levels of CarB (Fig 2B). Yp1945-L06 was excluded from the above average because its mutant *pepA* allele is present in only 30% of reads. Interestingly, point mutations observed at the extreme N-terminus of the protein, G8C and A16T, appear to exert a smaller influence on *carAB* expression than any other observed mutations (Fig 2).

### Zwf mutations may restore protein function

*Zwf* (YPO2066) encodes glucose-6-phosphate dehydrogenase, which is responsible for the conversion of glucose-6-phosphate to 6-phosphogluconolactone as the entrance to the pentose-phosphate pathway (PPP), and which serves as a modulator of PPP [60–62]. Zwf additionally functions in anabolic metabolism and in maintaining a reducing environment within the cell cytoplasm [63]. Zwf is inactive in *Y*. *pestis* due to a S155P mutation relative to the *Y*. *pseudotuberculosis* sequence [64, 65]. After passaging, *zwf* mutations were discovered in 4 populations derived from Yp1945 and 1 population from Yp2126 (Table 2). Three of the mutations found in this study alter position 155, including a reversion to serine in Yp1945-L10. Two mutations distal to position 155, H397Y and I382L, were also observed. Potential effects of these mutations are discussed below.

**Table 2:** Identified mutations in Zwf.

Population	Mutation
Yp1945-L01	P155T
Yp1945-L03	H397Y
Yp1945-L05	I382L
Yp1945-L10	P155S
Yp2126-L04	P155L

### pCD1 plasmid is a commonly lost genetic element

The pCD1 plasmid carries a number of genes encoding proteins required for virulence, including a number of outer membrane proteins [66] and supporting biosynthetic and secretory proteins [67], including those comprising a type 3 secretion system. Importantly, it also confers calcium-dependence during growth at 37°C [68]. After 60 passages at 28°C in BHI, only populations Yp1945-L10, Yp2126-L07, and Yp2126-L11 retained the pCD1 plasmid at high levels as judged by number of reads (185-, 308-, and 425-fold coverage as judged by analysis with MUMmer, respectively; data not shown). An additional two populations had low sequence coverage of pCD1: Yp1945-L04 and Yp2126-L09 had coverage of 12- and 17-fold coverage, respectively (data not shown). It is likely that there is strong selective pressure for loss of this plasmid, as low coverage levels were observed in multiple populations as early as passage 20 and complete loss was observed in most populations by passage 30. Plasmids pPCP1 and pMT1 were almost uniformly retained (data not shown).

### Large colony variants are a common occurrence in evolved populations

At the end of P60, cultures were serially diluted and plated onto BHI agar in order to directly measure cell density, which was not significantly different from that of the starting strains (data not shown). Although cell density was essentially unchanged at the end of the experiment, we were surprised to find colonies with markedly different morphologies than ancestral strains. Specifically, very large colonies were observed at high frequencies in almost all populations (S1 Table), with the notable exception of Yp1945-L06. We initially hypothesized that the large colony morphotype was due to loss of the *pgm* locus in these isolates, but close examination of sequence alignments revealed that lack of *pgm* is insufficient to explain this phenotype. Colony size variants after serial passage have been observed in *E*. *coli* and *Citrobacter freundii*. [15]. Large colony variants observed in this work are discussed in supporting information (S1 Table).

### Proteomic analysis of P60 cultures reveals common themes

Numerous proteins had significantly different abundances in evolved relative to ancestral strains (see S2 Spreadsheet for a complete list). Database searching as described above resulted in a total of 1074 identified proteins for Yp1945-derived populations and 1078 identified proteins for Yp2126-derived populations. Due to the stochastic nature of peptide/protein identification in shotgun proteomics (as opposed to mutation identification in genomics) we have not restricted our analysis to changes in protein abundance observed in individual lineages derived from both starting isolates. Instead we have identified proteins for which mean abundance ratios across all evolved lineages of a starting isolate have changed significantly from the mean abundance ratios across all replicate starting cultures, as described in Materials and Methods. Of the confidently identified proteins, 137 proteins in Yp1945-derived populations and 182 proteins in Yp2126-derived populations were present in significantly different abundances between P00 and P60 (a full listing of these proteins is found in the S2 Spreadsheet). We focus here on two of the broad functional categories of proteins classified using eggNOG [43]: 1) amino acid transport and metabolism, and 2) cell envelope and chaperones.

### Amino acid transport and metabolism

Numerous proteins identified as having involvement with amino acid transport and metabolism were expressed significantly differently in evolved vs. ancestral strains (Table 3). Of particular interest are two proteins responsible for the assimilation of amino nitrogen: GdhA and GlnA. *GdhA* (YPO3971) encodes glutamate dehydrogenase and is the key player in the glutamate dehydrogenase pathway of nitrogen metabolism. *GlnA* (YPO0024) encodes glutamine synthetase, the first step of the glutamine synthetase/glutamate synthase pathway. GdhA and GlnA were present at higher abundance in evolved lineages of both Yp1945 and Yp2126. In addition to glutamate and glutamine metabolism, protein abundance changes were observed in lysine, glycine, serine, and threonine metabolism (generally increased abundance) as well as phenylalanine, tyrosine, and tryptophan metabolism (generally decreased abundance). Increased CarAB protein abundance has been described above.

**Table 3:** Abundance ratios of proteins involved in amino acid metabolism.

Name	Function	Uniprot ID	Abundance Ratio 1945^1^	Abundance Ratio 2126^1^
argG/YPO1570	Argininosuccinate synthase	Q8ZFV7	AO/AO	NI/NI
argH/YPO3924	Argininosuccinate lyase	Q8ZA88	NI/NI	EO/EO
aroF/YPO3286	Phospho-2-dehydro-3-deoxyheptonate aldolase	Q0WC04	AO/AO	NI/NI
aroH/YPO2411	Phospho-2-dehydro-3-deoxyheptonate aldolase	Q7CIT5	AO/NI	AO/NI
artI/YPO4111	Periplasmic solute-binding protein	Q7CFN1	4.91/5.55	10.23/17.83
asnB/YPO2623	Asparagine synthetase	Q7CJU5	0.37/NI	0.49/NI
dapD/YPO1041	Tetrahydropicolinate succinylase	Q8ZH69	2.87/2.76	2.04/2.04
dapE/YPO3053	Succinyl-diaminopimelate desuccinylase	Q7CJI9	AO/NI	AO/AO
gcvP/YPO0905	Glycine dehydrogenase	Q8ZHI8	2.15/2.08	NI/NI
gcvT/YPO0907	Aminomethyltransferase	Q8ZHI6	2.09/NI	NI/NI
gdhA/YPO3971	Glutamate dehydrogenase	Q0WA46	11.47/NI	104.63/94.65
glnA/YPO0024	Glutamine synthetase	Q74YC3	5.90/NI	20.87/47.80
ilvC/YPO3888	Ketol-acid reductoisomerase	Q8ZAC2	2.48/2.38	NI/NI
iscS/YPO2896	Cysteine desulfrase	Q7CJN7	2.90/NI	NI/NI
pepA/YPO344	Leucyl aminopeptidase	Q8ZBH3	2.92/2.56	2.89/2.90
pepB/YPO2889	Aminopeptidase B	P58475	2.20/2.10	6.18/7.19
proV/YPO2647	Glycine betaine/L-proline transport ATP-binding protein	Q0WDN9	NI/NI	0.48/NI
selD/YPO2164	Selenophosphate synthase	Q8ZEK1	AO/AO	NI/NI
trpE/YPO2208	Anthranilate synthase component	Q7CIM3	NI/NI	AO/AO
ureA/YPO2665	Urease subunit alpha	P69994	2.38/2.32	4.63/4.56
ureB/YPO2666	Urease subunit beta	P69990	3.30/3.30	7.89/6.30
ureC/YPO2667	Urease subunit gamma	Q9ZFR9	2.772.70	4.17/4.11

^1^Abundance ratio reflects ratio of protein levels in evolved relative to ancestral populations.

NI, not identified (either protein was not identified during database search or abundance ratio did not meet significance threshold); EO, protein observed in evolved populations only; AO, protein observed in ancestral populations only.

Abundance ratios reflect “1 peptide”/”2 peptides” as described in discussion.

Interestingly, we observed substantially increased abundance of the urease holoenzyme (UreABC; YPO2665/2666/2667), despite the fact that *Y*. *pestis* is phenotypically urease-negative due to a single base insertion in the cryptic *ureD* gene [69]. Implications of this finding are discussed below.

Finally, we also investigated carbohydrate content of these populations during the course of this experiment. Although not a carbohydrate, the polyamine putrescine was readily detected by GC-MS in these samples. Putrescine levels were elevated in Yp1945- and Yp2126-derived lineages (1.41-fold higher, *p* = 1.45e^-5^; 2.01-fold higher, *p* = 1.08e^-7^, respectively). Polyamine production is related to amino acid production, and putrescine is a common intermediate in the metabolism of multiple amino acids.

### Envelope biogenesis and chaperones

Cell envelope biogenesis and chaperone activity are interconnected in bacteria, and so are reported here together (Table 4). In general, chaperone protein abundance is lower in evolved populations. In particular, DegP (YPO3382), IbpB (YPO4084), and RseP (YPO1051) are all involved in envelope stress response [70–72] and are present in lower abundance after laboratory evolution. In contrast, FkpA (YPO0195), GroL (YPO0351), Skp (YPO1053), and SlyD (YPO1093) are all primarily responsible for envelope biogenesis [73–76] and are present in higher abundance after laboratory evolution. SurA, thought to be the major periplasmic chaperone for outer membrane proteins (OMPs) in *E*. *coli* [75], does not follow this trend; it is only observed in ancestral populations of Yp2126.

**Table 4:** Abundance ratios of proteins involved in cell wall/membrane biogenesis and chaperones/folding factors.

Protein Name	Protein Function	UniProt ID	Expression Ratio 1945^1^	Expression Ratio 2126^1^
alr/YPO0321	Alanine racemase 1	Q8ZJ10	AO/AO	NI/NI
ampD/YPO1683	N-acetylmuramoyl-L-alanine amidase	Q7CIX7	NI/NI	0.47/0.48
clpB/YPO0506	ATP-dependent protease	Q7CG96	0.11/0.11	0.10/0.09
clpP/YPO3157	Endopeptidase Clp	Q8ZC65	NI/NI	EO/3.35
dacC/YPO1320	D-alanyl-D-alanine carboxypeptidase	Q7CHG0	2.05/2.92	2.32/2.43
degP/YPO33832	Periplasmic protease/chaperone	Q7CKD3	0.44/0.42	NI/NI
degQ/YPO3566	Protease	Q74PR7	NI/NI	0.35/0.37
fbaA/YPO0920	Fructose-bisphosphate aldolase class II	Q7CGS5	2.22/2.17	NI/NI
fkpA/YPO0195	Peptidyl-prolyl cis-trans isomerase	Q0WKB1	2.74/2.59	11.63/12.36
glnD/YPO1042	Protein-PII uridylyltransferase	Q8ZH68	NI/NI	AO/NI
groL/YPO0351	60 kDa chaperonin	Q8ZIY3	2.91/2.90	4.25/4.15
grxA/YPO1327	Glutaredoxin 1	Q7CHG3	NI/NI	2.16/NI
ibpB/YPO4084	Small heat shock protein IbpB	Q8Z9V6	NI/NI	AO/AO
lgt/YPO0784	Prolipoprotein diacylglyceryl transferase	Q8ZHV0	NI/NI	0.38/NI
lolB/YPO2015	Outer membrane lipoprotein LolB	Q8ZEY0	NI/NI	AO/AO
lpxD/YPO1054	UDP-3-O-(3-hydroxymyristoyl)glucosamine N-acyltransferase	P58611	NI/NI	AO/NI
mpl	Putative ligase	Q7CKK2	NI/NI	AO/AO
ompF/YPO1411	Outer membrane porin protein F	Q0WH04	15.48/14.74	24.93/18.17
ompW/YPO2201	Putative exported protein	Q0WEW6	EO/EO	NI/NI
rafQ/YPO0186	Putative LPS biosynthetic enzyme	Q3V8F4	NI/NI	AO/NI
rffG/YPO2602	dTDP-glucose 4,6-dehydratase	Q0WAE8	NI/NI	AO/NI
rlpA/YPO2602	Rare lipoprotein A	Q8ZDG6	AO/NI	NI/NI
rseP/YPO1051	Protease RseP	Q8ZH59	NI/NI	AO/AO
skp/YPO1053	Chaperone protein Skp	P58607	NI/NI	2.30/2.03
slyD/YPO0193	Metallochaperone SlyD	Q7CFU4	2.14/2.08	NI/NI
surA/YPO0494	Chaperone SurA	Q7CG87	NI/NI	AO/NI
trxB/YPO1374	Thioredoxin reductase	Q7CHI6	3.69/3.28	NI/2.14
wecB/YPO3864	UDP-N-acetylglucosamine 2-epimerase	Q8ZAE3	NI/NI	AO/NI
YPO2072	Uncharacterized protein	Q0WF84	NI/NI	EO/NI
YPO2155	Putative exported protein	Q7CIH1	NI/NI	0.50/NI
YPO2855	Putative protease	Q7CJL2	2.12/2.05	NI/NI
YPO3524	Putative exported protein	Q7CKK5	NI/NI	AO/NI

^1^Abundance ratio reflects ratio of protein levels in evolved relative to ancestral populations.

NI, not identified (either protein was not identified during database search or abundance ratio did not meet significance threshold);

EO, protein observed in evolved populations only;

AO, protein observed in ancestral populations only.

Abundance ratios reflect “1 peptide”/”2 peptides” as described in discussion.

While not themselves chaperones, LolB (YPO2015) and LpxD (YPO1054) are responsible for lipoprotein [77] and lipopolysaccharide (LPS) [78] localization in the OM and are only observed only in ancestral populations of Yp2126. OmpF (YPO1411), which is a major porin residing in the OM, is significantly more abundant after evolution in both Yp1945- and Yp2126-derived lineages. RseP, a critical component of the σ^E^ activation cascade, is only observed in ancestral Yp2126.

Proteins responsible for cell wall synthesis are also present in altered abundances in evolved populations: Alr (YPO0321) and AmpD (YPO1683) are significantly less abundant in evolved populations relative to ancestor strains (only observed in ancestral Yp1945 and expression ratio of 0.47 in Yp2126-derived populations, respectively). A notable exception to this pattern is DacC (YPO1320), an inner membrane penicillin-binding protein, which is present in significantly higher abundance after evolution.

## Discussion

### Genomic investigation of Y. pestis laboratory adaptation

Mutations in *ail* resulted universally in disruption or premature truncation of Ail protein (Table 1), and can be explained by invoking reduced metabolic load; since Ail is one of the most highly transcribed genes in Yersiniae [50, 51] and is not required for laboratory growth, reduced synthesis of the protein in the mutants should concomitantly increase the ability of mutant cells to produce other cellular components necessary to outcompete other members of the population. Pieper et al [48, 79] showed that Ail is more abundant at 37°C than at 26°C, but it is important to note that Ail is abundant in the outer membrane even at 26°C (see Fig 4 of reference [48] and Fig 1 of reference [79]). With the exception of Yp1945-L06, all lineages containing mutant *ail* have either low or undetectable levels of Ail protein after evolution. Yp1945-L05, Yp2126-L02, Yp2126-L07, Yp2126-L10, Yp2126-L11, and Yp2126-L12 are all genetically wild-type with respect to *ail*, but do not express Ail at detectable levels after laboratory evolution. This suggests that there is a significant role played by Ail downregulation independent of direct mutation. Since Ail is required for invasion of host cells, it is likely that populations lacking the protein have attenuated virulence, although this hypothesis was not tested here.

*PepA* mutations were dispersed throughout the protein, occurring in both the DNA-binding and aminopeptidase domains (see [52] for a discussion of *E*. *coli* PepA structure and function). Similar mutations have been observed in other *pepA* mutants; many of them resulted in increased *carAB* expression [52, 53], suggesting that the observed PepA mutants in our study also result in increased *carAB* transcription. Proteomic data show that expression of CarAB was significantly elevated in the presence of all of these mutations (Fig 2A and 2B). These data suggest that three distinct but related mechanisms could be responsible for de-repression of the carAB operon. First, point mutations in the DNA-binding domain likely alter PepA function and prevent repression of *carAB* transcription. Second, indels leading to premature truncation throughout the protein likely result in loss of the protein entirely. The third possible mechanism of derepression of *carAB* transcription is to prevent interaction between PepA monomers by altering amino acids at the C-terminus of the protein. C-terminal amino acids interact with N-terminal amino acids of adjoining PepA monomers during Xer site-specific recombination [80]. It is likely that prevention of monomer interactions hinders proper protein assembly and function. The fact that a particular mutation at the N-terminus of the protein, G8T, only modestly increases CarAB expression suggests that a modest increase in the pool of cellular carbamoylphosphate provides sufficient benefit to cells under these conditions. The data presented here suggest that increased levels of CarAB protein are of particular benefit to cells growing in rich media in the laboratory. This is supported by the observation that evolved populations expressing wild-type PepA also have slightly higher levels of CarA and CarB proteins relative to ancestral strains (Fig 2A and 2B), and also indicates that another mechanism or mechanisms may influence *carAB* transcription under these conditions.

Zwf is normally inactive in *Y*. *pestis* biovar Orientalis [65, 81] due to a mutation of S155P relative to *Y*. *pesudotuberculosis*. The observed mutations in this experiment, including a reversion to “wild-type” Zwf (i.e. P155S; Table 2) suggest that utilization of the oxidative steps of PPP is a selectable trait under these conditions. The ability of *Y*. *pestis* cells to effectively shunt the carbon contained in media glucose and cellular glucose-6-phosphate to biosynthetic pathways, i.e. PPP instead of TCA cycle, may confer an advantage during laboratory growth. We speculate that the observed P155S mutation enables use of PPP by restoring Zwf function, and it is possible that P155T and P155L mutations restore function as well, although probably to lower levels. The effects of the remaining mutations, H397Y and I382L, are not easy to predict. Cleavage of Zwf by ClpXP cytoplasmic protease produces extracellular death factor (EDF) [82], amplifying the activities of MazEF and ChpBK toxins [83]. It is unlikely that H397Y or I382L affect EDF production (positions 199–203; [82]), although they may alter Zwf protein folding in such a way as to overcome presumable misfolding caused by the presence of proline instead of serine at position 155.

Plasmid loss has been observed in other laboratories after passaging [84, 85]. Loss of the pCD1 plasmid may also be favored under the conditions of our experiment: pCD1 contains the genes for the low calcium response (LCR; [86]), which is not invoked at temperatures below 37°C. As our PSPE was performed at 28°C it is likely that due to a lack of selection for maintenance of the LCR, cells were cured of this plasmid and thus escaped the metabolic cost of maintenance. In contrast, plasmids pMT1 and pPCP1 were maintained during laboratory passage. Maintenance of pMT1 is likely related to the high expression of murine toxin (S2 Spreadsheet); the phospholipase activity of murine toxin may be advantageous in BHI and could therefore provide selective pressure to maintain this plasmid. Pesticin and the pesticin immunity protein are encoded by pPCP1. Toxin/antitoxin systems often consist of a stable toxin and labile antitoxin [87, 88], and although relative stability of the pesticin/pesticin immunity proteins has not been investigated it is likely that they follow the same pattern. Pesticin is expressed at detectable levels in evolved populations; it therefore follows that cells within the population must maintain pPCP1 in order to also express the immunity protein.

Pervasive loss of the *pgm* locus in populations derived from Yp1945 may be a completely neutral event not requiring selection. *Pgm* is bounded by IS*100* elements, and is commonly lost during laboratory culture [89]. Nevertheless, a model in which *pgm* is lost due to neutral events is insufficient to explain the fact that Yp2126-derived populations uniformly maintained the *pgm* locus. Further investigation into this phenomenon is warranted.

### Proteomic investigation of *Y*. *pestis* laboratory adaptation

In contrast to the genomic analysis above, in which we focused on specific mutations present in lineages derived from both starting strains, proteomic analysis of evolved populations took a wider view and examined broad categories of proteins differing significantly in abundance from ancestral strains. We took this approach largely due to the stochastic nature of shotgun proteomics; inefficiencies in peptide detection and identification during LC-MS/MS can ultimately result in a less than complete biological picture for any one biological replicate, even with multiple technical replicates. In this experiment, shotgun proteomics provided a high-level view of metabolic processes, and in some cases provided data to support or disprove hypotheses generated by genomic data. In addition to the initial analysis using a minimum of one peptide to identify a protein, we refined our analysis to require two peptides for identification. This increased stringency did not qualitatively affect our results. Most protein quantification was unaffected. The majority of protein quantification that was affected was due to subtle differences resulting in abundance changes that only just failed to meet our search criteria. On their own, proteomic data generated during this experiment have shown promising avenues for further hypothesis-driven research independent of observing specific mutations.

Alteration of cell envelope constituents is a key result of laboratory adaptation in this study (Table 4). In this study, we observed downregulation of key stress response proteins. DegP is the major periplasmic protease [90], and is thought to be responsible for rescuing OMPs that have fallen off the SurA-based assembly pathway, in concert with Skp [75]. DegP levels are reduced after laboratory adaptation, as are SurA levels, suggesting a low overall flux of OMP intermediates through the envelope. As Skp can function to rescue *degP-* cells under certain conditions [75], it is not surprising that Skp and DegP protein abundances follow opposite trends. Further support for low overall OMP flux through the cell envelope comes from low levels of RseP in evolved lineages. RseP functions to liberate the alternative sigma factor σ^E^ from the membrane protein RseA during envelope stress response [91], and from low RseP protein levels we infer that envelope stress is low. Lower abundance of additional envelope assembly factors after laboratory adaptation was also common. We observed lower levels of LolB and LpxD in adapted strains. These genes are responsible for proper localization of OM lipoproteins [77] and LPS [78]. LpxD is itself localized in the OM, again highlighting low overall OMP flux through the cell envelope. It is important to note here that both LolB and LpxD are essential in *E*. *coli*; therefore it is not possible to infer complete cellular absence from a lack of identification of these or other proteins in this discussion using proteomics. Overall, our observations of cell envelope proteins support the assertion that adapted cells experience far lower stress in the laboratory growth environment (in this case, BHI broth) than their wild ancestors.

We observed significantly elevated levels of the UreABC urease holoenzyme. Intriguingly, *Y*. *pestis* is phenotypically urease-negative due to a single base insertion in the cryptic *ureD* gene, which introduces a premature stop codon [69] in the metallochaperone protein required for inserting a nickel ion to form a mature holoenzyme. Since a reversion of *ureD* is possible, and since we observed elevated UreABC levels, we sequenced *ureD* using Sanger sequencing and tested laboratory-adapted strains using an agar-based urease assay (Beckton-Dickenson). Neither a reversion to full-length *ureD* nor urease-positive phenotypes were observed. However, our proteomic results unambiguously show that the *ureABC* genes are not only transcribed and translated, but also are upregulated during long-term laboratory cultivation on BHI.

The power of combining genomic with proteomic analyses was particularly highlighted when we examined the connection between mutant PepA and elevated putrescine levels. CarAB catalyzes the conversion of L-glutamine to L-glutamate and carbamoylphosphate. Carbamoylphosphate can enter the urea cycle by reacting with ornithine to form arginine. Because the urea cycle in *Y*. *pestis* is blocked by the lack of arginase, the cells convert arginine to agmatine via arginine decarboxylase. Agmatine is ultimately converted to putrescine by the successive action of agmatine deaminase and N-carbamoyl putrescine decarboxylase. We hypothesize that *Y*. *pestis* cells use putrescine and possibly other polyamine compounds as nitrogen sinks during growth on amino nitrogen-rich compounds, and that this use drives the fixation of mutations such as we observed in *pepA* (Fig 2). This fits generally with the differences between BHI and mammalian/flea host environments, although the lack of knowledge of basic *Y*. *pestis* physiology leaves some of the details unclear. BHI is particularly rich in nitrogen, containing 0.31 moles nitrogen per 1 mole carbon, and after growth in BHI this ratio is further decreased in *Y*. *pestis* biomass (Kreuzer, unpublished data). Nitrogen in BHI is supplied mostly in the form of oligopeptides and amino acids. Since glucose is rapidly depleted during exponential phase growth, cells growing on BHI would presumably need to use amino acids as a carbon source by the time the culture reaches stationary phase. It is therefore reasonable to speculate that an increased ability to use amino acid carbon backbones for energy and biomass by stripping them of their amino groups would be advantageous in BHI. Free amines could then ultimately be stored as polyamine compounds such as putrescine.

This PSPE showed that alterations in global nitrogen metabolism, especially alterations in amino acid biosynthesis, are key adaptations to growth in the laboratory (Table 3). Mechanisms of global nitrogen metabolism in enterobacteria have been described, although they come primarily from studies of *E*. *coli*, *Klebsiella*, and *Salmonella* [92, 93], and are not well understood in *Y*. *pestis*. *Y*. *pestis* differs fundamentally from *E*. *coli* and *Klebsiella*. First *Y*. *pestis* does not possess the nitrogen assimilation control (NAC) gene, which encodes a secondary transcription factor implicated in global response to intracellular nitrogen [92]. A BLAST search of the entire Uniprot database using *K*. *pneumonia* NAC failed to yield any hits to any *Yersinia* species. Second, *Y*. *pestis* lacks several key enzymes active in the downstream metabolism of amino acids and nitrogenous compounds: aspartase, arginase, and urease. Thus, models of nitrogen metabolism built using other enterobacteria may not be fully applicable when interpreting data from *Y*. *pestis*. Future work should be geared towards closing this knowledge gap.

## Supporting Information

S1 Spreadsheet Genomic mutations in laboratory-evolved *Y*. *pestis* populations derived from ancestor strains Yp1945 and Yp2126. Provided as a fully searchable Microsoft Excel spreadsheet with multiple tabs. *Read evidence* tabs provide the fraction of reads in each evolved population exhibiting the indicated mutation. Green, yellow, and red highlighting indicate ≥75%, between 20% and 75%, 5%-20% of reads for a given population exhibit the indicated mutation. *Missing coverage evidence* tabs summarize genes for which evolved populations were missing read coverage, indicating genomic deletions. *New junction evidence* tabs detail the reads providing evidence for novel junctions as a complement to the missing coverage information.
(XLSX)

S2 Spreadsheet Proteomics results for ancestral Yp1945 and Yp2126 strains and their respective laboratory-evolved descendent populations. Data are the normalized, log2-transformed protein abundance values derived from LC-MS/MS experiments as reported by the MaxQuant analysis software. Each column labeled” P00.x” (where *x* is an identifier, 1–10 or A, B) represents an independent biological replicate (average of three or more injection replicates) of the ancestral culture. Each column labeled “L*y*” (where y identifies the parallel cultures from 1–12) represents an independently evolved (60 passages) population, (average of injection replicates). “q” is the q-value calculated by Inferno. “Unique P00” and “Unique P60” describe whether a given protein is observed exclusively in one or the other conditions, with 1 indicating logical “true” and 0 “false” (see text for details). “Log2 Fold Change” is the overall expression ratio, log2-transformed. “Final Filter” indicates (1 = true, 0 = false) whether the given protein meets all of the criteria for consideration as significantly changing. See Methods section for details.
(XLSX)

S1 Table Large colony phenotype in evolved strains and relationship to loss of pCD1 and *pgm* loci. Provided as a Microsoft Word document. The table records the frequency of the large colony phenotype and the loss or maintenance of the two loci. The image shows an example of the large colony morphotype together with wild-type colonies. Supplemental text describes the large colony phenotype.
(DOCX)

S2 Table SNPs and indels present in the ancestral Yp1945 strain relative to *Y*. *pestis* CO92. The small number of differences shows that Yp1945 is closely related to CO92.
(DOCX)

S3 Table SNPs and indels present in the ancestral Yp2126 strain relative to *Y*. *pestis* CO92. The small number of differences shows that Yp2126 is closely related to CO92.
(DOCX)
