# Supplementary material for: Investigation of Yersinia pestis Laboratory Adaptation through a Combined Genomics and Proteomics Approach
Source: PLoS One. 2015 Nov 24;10(11):e0142997. doi: 10.1371/journal.pone.0142997 (PMC4658026; doi:10.1371/journal.pone.0142997)
Supplement: S1 Table — Provided as a Microsoft Word document. The table records the frequency of the large colony phenotype and the loss or maintenance of the two loci. The image shows an example of the large colony morphotype together with wild-type colonies. Supplemental text describes the large colony phenotype. (DOCX) [file pone.0142997.s003.docx]

| **Population^1^** | **Large Colony Frequency** | **pCD1** | ***pgm*** |
| --- | --- | --- | --- |
| Yp1945-L01 | 100.0% | - | - |
| Yp1945-L02 | 100.0% | - | - |
| Yp1945-L03 | 97.9% | - | - |
| Yp1945-L04 | 71.1% | +/- | - |
| Yp1945-L05 | 100% | - | - |
| Yp1945-L06 | 0.0% | - | + |
| Yp1945-L07 | 93.2% | - | - |
| Yp1945-L08 | 53.4% | - | - |
| Yp1945-L09 | 51.0% | - | - |
| Yp1945-L10 | 100.0% | + | - |
| Yp1945-L11 | 64.3% | - | - |
| Yp1945-L12 | 97.8% | - | - |
| Yp2126-L02 | 55.6% | - | + |
| Yp2126-L03 | 74.5% | - | + |
| Yp2126-L04 | 92.9% | - | + |
| Yp2126-L05 | 38.1% | - | + |
| Yp2126-L06 | 72.4% | - | + |
| Yp2126-L07 | 100.0% | + | + |
| Yp2126-L08 | 86.2% | - | + |
| Yp2126-L09 | 98.3% | +/- | + |
| Yp2126-L10 | 98.8% | - | + |
| Yp2126-L11 | 88.1% | + | + |
| Yp2126-L12 | 66.0% | - | + |
| ^1^Yp2126-L01 was contaminated early in the experiment and was discarded from further analyses | | | |


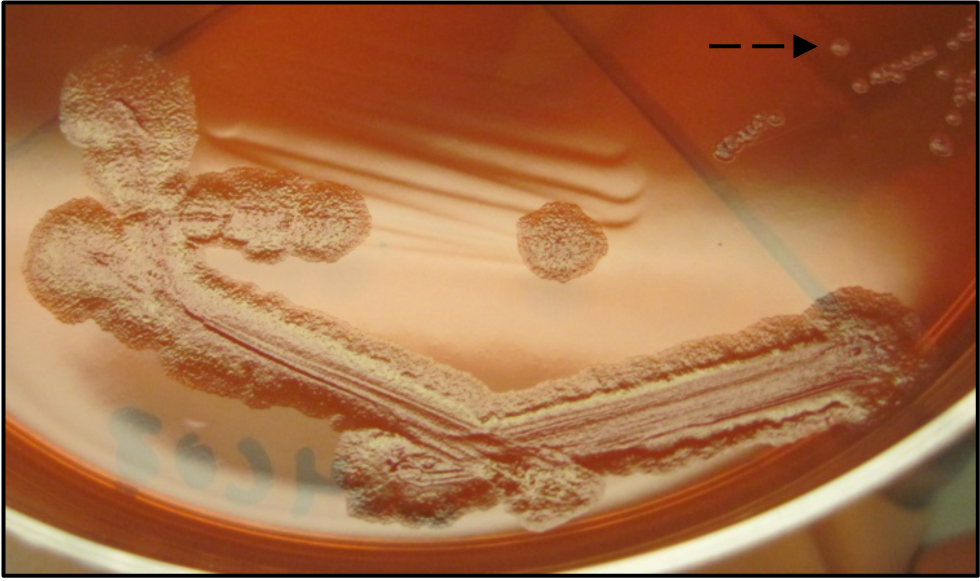


Example of large colony morphotype after 60 passages. Large colonies were approximately 5-6 mm after 48 h growth at 28 °C. Dashed arrow indicates ancestral (wild-type) colony, approximately 1-2 mm in diameter after the same period.

In this study, we found that a large-colony phenotype commonly arose in the passaged populations. Wild-type *Y. pestis* cells, both the starting strains used in this study and other strains, normally grow to approximately 1-2 mm on blood agar plates at 26 °C after 48 h, whereas our evolved populations typically contained a significant proportion of large (>5-6 mm) colonies when incubated under the same conditions (Supplemental Material). It is not clear which mutation(s) contribute to this phenotype in *Y. pestis*. There was no correlation between loss of the *pgm* locus and large colony size. Similarly, there was no consistent set of mutated genes across populations exhibiting the phenotype, nor was there a consistent set of significantly changing proteins. Interestingly, colony size and morphology variants have also been observed after serial passaging of wild *E. coli* and *Citrobacter* spp. in laboratory media [[15](#_ENREF_15)]. It would be interesting to determine whether a common mechanism results in large-colony morphotypes in these three different Enterobacteria.
