# Supplementary material for: Investigation of Yersinia pestis Laboratory Adaptation through a Combined Genomics and Proteomics Approach
Source: PLoS One. 2015 Nov 24;10(11):e0142997. doi: 10.1371/journal.pone.0142997 (PMC4658026; doi:10.1371/journal.pone.0142997)
Supplement: S2 Table — The small number of differences shows that Yp1945 is closely related to CO92. (DOCX) [file pone.0142997.s004.docx]

| **SNPs present in Yp1945 relative to YpCO92** | | | | | | |
| --- | --- | --- | --- | --- | --- | --- |
| **Location** | **Position** | **Mutation** | **Read Frequency** | **Annotation**^1^ | **Gene**^2^ | **Gene Product Description** |
| pMT1 | 62,994 | T→C | 100.0% | intergenic (‑205/‑3) | *YPMT1.61c* ← / → *YPMT1.61N* | antirestriction protein/hypothetical protein |
| Chromosome | 82 | C→A | 100.0% | intergenic (–/+189) | – / ← *YPO0001* | –/flavodoxin |
| Chromosome | 130 | G→C | 100.0% | intergenic (–/+141) | – / ← *YPO0001* | –/flavodoxin |
| Chromosome | 120,716 | C→T | 100.0% | intergenic (+529/+32) | *rpmE* → / ← *YPO0112* | 50S ribosomal protein L31/hypothetical protein |
| Chromosome | 120,719 | C→T | 100.0% | intergenic (+532/+29) | *rpmE* → / ← *YPO0112* | 50S ribosomal protein L31/hypothetical protein |
| Chromosome | 120,722 | T→C | 100.0% | intergenic (+535/+26) | *rpmE* → / ← *YPO0112* | 50S ribosomal protein L31/hypothetical protein |
| Chromosome | 150,946 | C→A | 100.0% | T524T (ACG→ACT) | *pckA* ← | phosphoenolpyruvate carboxykinase |
| Chromosome | 351,821 | T→G | 100.0% | H617Q (CAT→CAG) | *YPO0342* → | oxidoreductase Fe‑S binding subunit |
| Chromosome | 845,472 | C→T | 92.4% | A1051T (GCA→ACA) | *YPO0776* ← | siderophore biosysnthesis protein |
| Chromosome | 845,499 | T→C | 93.8% | T1042A (ACA→GCA) | *YPO0776* ← | siderophore biosysnthesis protein |
| Chromosome | 917,155 | A→G | 100.0% | S60G (AGT→GGT) | *YPO0837* → | PTS permease |
| Chromosome | 1,178,178 | T→C | 100.0% | intergenic (+219/‑35) | *tnp* → / → *YPO1037* | transposase for the IS1541 insertion element/hypothetical protein |
| Chromosome | 1,290,208 | C→A | 92.2% | intergenic (‑110/‑207) | *modE* ← / → *YPO1144* | DNA‑binding transcriptional regulator ModE/hypothetical protein |
| Chromosome | 1,391,153 | C→T | 97.6% | intergenic (‑193/+229) | *cheD2* ← / ← *pla2* | methyl‑accepting chemotaxis protein/outer membrane protease |
| Chromosome | 1,448,300 | G→A | 96.3% | intergenic (‑208/‑144) | *YPO1289* ← / → *YPO1290* | hypothetical protein/aldehyde dehydrogenase |
| Chromosome | 1,459,291 | G→A | 97.4% | R72H (CGC→CAC) | *fruA* → | PTS system fructose‑specific transporter subunits IIBC |
| Chromosome | 1,691,290 | C→T | 100.0% | intergenic (‑527/‑156) | *YPO1490* ← / → *ybiT* | hypothetical protein/ABC transporter ATP‑binding protein |
| Chromosome | 1,734,560 | C→A | 97.3% | P73H (CCT→CAT) | *YPO1526* → | assembly protein |
| Chromosome | 1,791,460 | T→C | 96.5% | intergenic (‑145/‑149) | *argG* ← / → *yeeO* | argininosuccinate synthase/hypothetical protein |
| Chromosome | 1,802,069 | G→T | 93.3% | intergenic (‑156/‑367) | *YPO1579* ← / → *YPO1580* | C4‑dicarboxylate transporter,periplasmic protein/phosphopantetheinyl transferase |
| Chromosome | 1,939,828 | T→G | 100.0% | A559A (GCA→GCC) | *YPO1701* ← | hypothetical protein |
| Chromosome | 1,939,828 | T→G | 100.0% | A559A (GCA→GCC) | *YPO1701* ← | hypothetical protein |
| 1 Numbers in parentheses refer to distance upstream or downstream of nearest gene  2 Arrows refer to gene orientation | | | | | | |

| **SNPs present in Yp1945 relative to YpCO92** | | | | | | |
| --- | --- | --- | --- | --- | --- | --- |
| **Location** | **Position** | **Mutation** | **Read Frequency** | **Annotation**^1^ | **Gene**^2^ | **Gene Product Description** |
| Chromosome | 1,939,841 | A→G | 100.0% | L555P (CTT→CCT) | *YPO1701* ← | hypothetical protein |
| Chromosome | 1,999,488 | C→T | 95.6% | G140S (GGC→AGC) | *yebN* ← | hypothetical protein |
| Chromosome | 2,158,063 | A→C | 97.3% | V1365G (GTG→GGG) | *irp2* ← | yersiniabactin biosynthetic protein |
| Chromosome | 2,273,616 | G→C | 100.0% | T50R (ACG→AGG) | *YPO2000* ← | two‑component system sensor protein |
| Chromosome | 2,278,317 | A→G | 100.0% | V40A (GTT→GCT) | *YPO2005* ← | hypothetical protein |
| Chromosome | 2,300,659 | T→G | 100.0% | D252A (GAC→GCC) | *YPO2029* ← | hypothetical protein |
| Chromosome | 2,619,611 | T→G | 100.0% | E56A (GAG→GCG) | *YPO2328* ← | hypothetical protein |
| Chromosome | 2,968,425 | A→G | 100.0% | intergenic (‑41/+8) | *YPO2640* ← / ← *intA* | IS1400 transposase A/phage family integrase (partial) |
| Chromosome | 3,128,420 | G→A | 95.7% | F14F (TTC→TTT) | *YPO2798* ← | hypothetical protein |
| Chromosome | 3,224,885 | G→A | 96.9% | E483E (GAG→GAA) | *yapA* → | autotransporter protein |
| Chromosome | 3,608,932 | T→C | 100.0% | D109G (GAC→GGC) | *gmhA* ← | phosphoheptose isomerase |
| Chromosome | 3,647,867 | C→T | 100.0% | A347V (GCT→GTT) | *pssA* → | phosphatidylserine synthase |
| Chromosome | 3,655,609 | T→C | 100.0% | K553E (AAA→GAA) | *clpB* ← | protein disaggregation chaperone |
| Chromosome | 3,739,401 | C→A | 100.0% | G70G (GGG→GGT) | *ydjJ* ← | Zinc‑binding dehydrogenase |
| Chromosome | 3,886,839 | T→C | 100.0% | E468E (GAA→GAG) | *ibeB* ← | outer membrane efflux lipoprotein |
| Chromosome | 4,219,380 | G→C | 100.0% | noncoding (1877/2907 nt) | *YPOr15* ← | 23S ribosomal RNA |
| Chromosome | 4,268,351 | C→A | 100.0% | intergenic (+30/‑83) | *YPO3801* → / → *YPO3802* | hypothetical protein/hypothetical protein |
| Chromosome | 4,268,358 | C→A | 100.0% | intergenic (+37/‑76) | *YPO3801* → / → *YPO3802* | hypothetical protein/hypothetical protein |
| Chromosome | 4,579,183 | A→G | 100.0% | S93G (AGC→GGC) | *fdhD* → | formate dehydrogenase accessory protein |
| Chromosome | 4,619,743 | C→T | 97.4% | intergenic (‑14/‑110) | *dnaA* ← / → *YPO4098* | chromosome replication initiator DnaA/hypothetical protein |
| Chromosome | 4,624,135 | C→G | 100.0% | P391R (CCC→CGC) | *trmE* → | tRNA modification GTPase TrmE |
| 1 Numbers in parentheses refer to distance upstream or downstream of nearest gene  2 Arrows refer to gene orientation | | | | | | |

| **Indels present in Yp1945 relative to YpCO92** | | | | | | |
| --- | --- | --- | --- | --- | --- | --- |
| **Location** | **Position** | **Mutation** | **Read Frequency** | **Annotation^1^** | **Gene^2^** | **Gene Product Description** |
| pCD1 | 5,407 | +A | 100.0% | intergenic (‑474/+788) | *YPCD1.09c* ← / ← *sopB* | hypothetical protein/plasmid‑partitioning protein |
| Chromosome | 17 | Δ1 bp | 100.0% | intergenic (–/+254) | – / ← *YPO0001* | –/flavodoxin |
| Chromosome | 27,834 | Δ20 bp | 96.8% | intergenic (‑346/‑380) | *engB* ← / → *YPO0020* | ribosome biogenesis GTP‑binding protein YsxC/hypothetical protein |
| Chromosome | 126,994 | 8 bp x 2 | 93.4% | duplication | *metF* → / ← *YPO0118* | 5,10‑methylenetetrahydrofolate reductase/transposase for insertion sequence IS1661 |
| Chromosome | 322,216 | 7 bp x 2 | 95.0% | duplication | *lexA* → / ← *YPO0315* | LexA repressor/zinc uptake transcriptional repressor |
| Chromosome | 581,519 | +TTCAA | 97.9% | intergenic (+299/‑30) | *leuO* → / → *YPO0536* | leucine transcriptional activator/hypothetical protein |
| Chromosome | 808,087 | Δ7 bp | 96.8% | intergenic (+26/+846) | *YPO0751* → / ← *YPO0754* | hypothetical protein/hypothetical protein |
| Chromosome | 1,234,971 | +A | 100.0% | coding (220/312 nt) | *YPO1087* → | prophage protein |
| Chromosome | 1,315,935 | +27 bp | 91.7% | coding (834/2022 nt) | *betT* → | choline transport protein BetT |
| Chromosome | 1,395,956 | +18 bp | 90.9% | coding (33/612 nt) | *YPO1236* → | aldolase |
| Chromosome | 1,553,969 | Δ16 bp | 97.7% | deletion | *serS* | seryl-tRNA synthetase |
| Chromosome | 1,607,059 | 9 bp x 2 | 92.7% | duplication | *pepN* → / → *pyrD* | aminopeptidase/dihydroorotate dehydrogenase 2 |
| Chromosome | 1,792,310 | Δ1 bp | 95.0% | coding (702/1431 nt) | *yeeO* → | hypothetical protein |
| Chromosome | 1,868,887 | Δ16 bp | 97.4% | intergenic (‑222/+39) | *cscR* ← / ← *YPO1643* | sucrose operon repressor LacI family/hypothetical protein |
| Chromosome | 2,077,747 | +32 bp | 90.2% | intergenic (+62/‑200) | *fliE* → / → *YPO1832* | flagellar hook‑basal body protein FliE/hypothetical protein |
| Chromosome | 2,117,881 | 9 bp x 2 | 92.2% | duplication | *YPO1871* ← | hypothetical protein |
| Chromosome | 2,325,276 | Δ8 bp | 97.7% | intergenic (‑453/‑163) | *argS* ← / → *YPO2047* | arginyl‑tRNA synthetase/hypothetical protein |
| Chromosome | 2,415,050 | +A | 95.5% | intergenic (‑199/‑266) | *YPO2145* ← / → *dadA* | SpoVR family protein/D‑amino acid dehydrogenase small subunit |
| Chromosome | 2,552,609 | +G | 100.0% | intergenic (+47/+94) | *bioD* → / ← *YPO2270* | dithiobiotin synthetase/voltage‑gated ClC‑type chloride channel ClcB |
| Chromosome | 2,552,628 | Δ1 bp | 100.0% | intergenic (+66/+75) | *bioD* → / ← *YPO2270* | dithiobiotin synthetase/voltage‑gated ClC‑type chloride channel ClcB |
| Chromosome | 2,578,007 | 14 bp x 2 | 91.4% | duplication | *YPO2292* ← / → *ilvB* | lipoprotein/acetolactate synthase catalytic subunit |
| Chromosome | 2,769,622 | Δ60 bp | 98.2% | deletion | *YPO2469* | hypothetical protein |
| Chromosome | 2,771,484 | Δ6 bp | 92.2% | intergenic (+100/+478) | *YPO2470* → / ← *YPO2471* | hypothetical protein/hypothetical protein |
| Chromosome | 2,853,322 | 7 bp x 2 | 90.4% | duplication | *YPO2541* → / ← *YPO2542* | hypothetical protein/hypothetical protein |
| Chromosome | 2,895,310 | +C | 97.1% | intergenic (‑382/+307) | *YPO2574* ← / ← *YPO2576* | hypothetical protein/hypothetical protein |
| Chromosome | 3,132,285 | Δ1 bp | 93.6% | coding (2128/2208 nt) | *YPO2801* → | hypothetical protein |
| Chromosome | 3,208,218 | Δ24 bp | 99.8% | deletion | *xseA* | exodeoxyribonuclease VII large subunit |
| 1 Numbers in parentheses refer to distance upstream or downstream of nearest gene  2 Arrows refer to gene orientation | | | | | | |
| **Indels present in Yp1945 relative to YpCO92** | | | | | | |
| **Location** | **Position** | **Mutation** | **Read Frequency** | **Annotation^1^** | **Gene^2^** | **Gene Product Description** |
| Chromosome | 3,225,581 | Δ54 bp | 94.6% | coding (2145‑2198/4293 nt) | *yapA* → | autotransporter protein |
| Chromosome | 3,312,496 | +AATTA | 94.2% | intergenic (‑485/‑346) | *YPO2963* ← / → *dmsA* | hypothetical protein/dimethyl sulfoxide reductase chain A protein |
| Chromosome | 3,458,682 | 7 bp x 2 | 96.4% | duplication | *manC* ← / ← *wbyK* | mannose‑1‑phosphate guanylyltransferase/mannosyltransferase |
| Chromosome | 3,471,435 | Δ1 bp | 94.6% | intergenic (‑960/+138) | *ddhC* ← / ← *ddhA* | CDP‑4‑keto‑6‑deoxy‑D‑glucose‑3‑dehydratase/glucose‑1‑phosphate cytidylyltransferase |
| Chromosome | 3,618,871 | Δ6 bp | 95.9% | coding (1729‑1734/5733 nt) | *hmwA* ← | adhesin |
| Chromosome | 3,696,792 | Δ5 bp | 94.1% | intergenic (‑164/+88) | *YPO3314* ← / ← *rbsC* | N‑terminal region of transketolase/sugar ABC transporter permease |
| Chromosome | 3,743,969 | 8 bp x 2 | 92.1% | duplication | *mutS* → / ← *rpoS* | DNA mismatch repair protein MutS/RNA polymerase sigma factor RpoS |
| Chromosome | 4,000,718 | Δ1 bp | 100.0% | intergenic (+67/+3) | *YPO3592* → / ← *YPO3592A* | transposase/IS protein/insertion element IS1661 protein (gene fragment) |
| Chromosome | 4,296,703 | 8 bp x 2 | 92.3% | duplication | *YPO3828* ← | hypothetical protein |
| Chromosome | 4,457,747 | 8 bp x 2 | 90.5% | duplication | *YPO3960* ← | hypothetical protein |
| Chromosome | 4,610,606 | 8 bp x 2 | 91.2% | duplication | *YPO4090* → / → *YPO4091* | ornithine cyclodeaminase/insertion element IS1661 DNA‑binding protein |
| 1 Numbers in parentheses refer to distance upstream or downstream of nearest gene  2 Arrows refer to gene orientation | | | | | | |
