# Supplementary material for: Investigation of Yersinia pestis Laboratory Adaptation through a Combined Genomics and Proteomics Approach
Source: PLoS One. 2015 Nov 24;10(11):e0142997. doi: 10.1371/journal.pone.0142997 (PMC4658026; doi:10.1371/journal.pone.0142997)
Supplement: S3 Table — The small number of differences shows that Yp2126 is closely related to CO92. (DOCX) [file pone.0142997.s005.docx]

| **SNPs present in Yp2126 relative to YpCO92** | | | | | | |
| --- | --- | --- | --- | --- | --- | --- |
| **Location** | **Position** | **Mutation** | **Read Frequency** | **Annotation^1^** | **Gene^1^** | **Gene Product Description** |
| pMT1 | 62,994 | T→C | 100.0% | intergenic (‑205/‑3) | *YPMT1.61c* ← / → *YPMT1.61N* | antirestriction protein/hypothetical protein |
| Chromosome | 82 | C→A | 100.0% | intergenic (–/+189) | – / ← *YPO0001* | –/flavodoxin |
| Chromosome | 130 | G→C | 100.0% | intergenic (–/+141) | – / ← *YPO0001* | –/flavodoxin |
| Chromosome | 120,716 | C→T | 100.0% | intergenic (+529/+32) | *rpmE* → / ← *YPO0112* | 50S ribosomal protein L31/hypothetical protein |
| Chromosome | 120,719 | C→T | 100.0% | intergenic (+532/+29) | *rpmE* → / ← *YPO0112* | 50S ribosomal protein L31/hypothetical protein |
| Chromosome | 120,722 | T→C | 100.0% | intergenic (+535/+26) | *rpmE* → / ← *YPO0112* | 50S ribosomal protein L31/hypothetical protein |
| Chromosome | 150,946 | C→A | 100.0% | T524T (ACG→ACT) | *pckA* ← | phosphoenolpyruvate carboxykinase |
| Chromosome | 351,821 | T→G | 100.0% | H617Q (CAT→CAG) | *YPO0342* → | oxidoreductase Fe‑S binding subunit |
| Chromosome | 877,843 | C→T | 100.0% | G367E (GGG→GAG) | *YPO0798* ← | sugar transport protein |
| Chromosome | 917,155 | A→G | 100.0% | S60G (AGT→GGT) | *YPO0837* → | PTS permease |
| Chromosome | 1,178,178 | T→C | 100.0% | intergenic (+219/‑35) | *tnp* → / → *YPO1037* | transposase for the IS1541 insertion element/hypothetical protein |
| Chromosome | 1,343,829 | G→A | 100.0% | intergenic (+76/+79) | *YPO1193* → / ← *YPO1194* | pyridoxal‑dependent decarboxylase/sulfurtransferase |
| Chromosome | 1,343,836 | A→G | 100.0% | intergenic (+83/+72) | *YPO1193* → / ← *YPO1194* | pyridoxal‑dependent decarboxylase/sulfurtransferase |
| Chromosome | 1,771,778 | G→T | 100.0% | D4Y (GAT→TAT) | *yeeF* → | amino acid permease |
| Chromosome | 1,939,828 | T→G | 100.0% | A559A (GCA→GCC) | *YPO1701* ← | hypothetical protein |
| Chromosome | 1,939,841 | A→G | 100.0% | L555P (CTT→CCT) | *YPO1701* ← | hypothetical protein |
| Chromosome | 2,204,699 | T→G | 100.0% | intergenic (+53/‑248) | *YPO1942* → / → *YPO1943* | hypothetical protein/hypothetical protein |
| Chromosome | 2,204,721 | T→G | 100.0% | intergenic (+75/‑226) | *YPO1942* → / → *YPO1943* | hypothetical protein/hypothetical protein |
| Chromosome | 2,204,742 | T→G | 100.0% | intergenic (+96/‑205) | *YPO1942* → / → *YPO1943* | hypothetical protein/hypothetical protein |
| Chromosome | 2,204,770 | T→A | 100.0% | intergenic (+124/‑177) | *YPO1942* → / → *YPO1943* | hypothetical protein/hypothetical protein |
| Chromosome | 2,204,773 | C→G | 100.0% | intergenic (+127/‑174) | *YPO1942* → / → *YPO1943* | hypothetical protein/hypothetical protein |
| Chromosome | 2,273,616 | G→C | 100.0% | T50R (ACG→AGG) | *YPO2000* ← | two‑component system sensor protein |
| Chromosome | 2,278,317 | A→G | 100.0% | V40A (GTT→GCT) | *YPO2005* ← | hypothetical protein |
| Chromosome | 2,300,659 | T→G | 100.0% | D252A (GAC→GCC) | *YPO2029* ← | hypothetical protein |
| 1 Numbers in parentheses refer to distance upstream or downstream of nearest gene  2 Arrows refer to gene orientation | | | | | | |

| **SNPs present in Yp2126 relative to YpCO92** | | | | | | |
| --- | --- | --- | --- | --- | --- | --- |
| **Location** | **Position** | **Mutation** | **Read Frequency** | **Annotation^1^** | **Gene^1^** | **Gene Product Description** |
| Chromosome | 2,444,561 | T→G | 100.0% | intergenic (‑842/‑492) | *YPO2172* ← / → *YPO2173* | hypothetical protein/response regulator of RpoS |
| Chromosome | 2,619,611 | T→G | 100.0% | E56A (GAG→GCG) | *YPO2328* ← | hypothetical protein |
| Chromosome | 2,968,425 | A→G | 100.0% | intergenic (‑41/+8) | *YPO2640* ← / ← *intA* | IS1400 transposase A/phage family integrase (partial) |
| Chromosome | 3,207,901 | G→A | 100.0% | V351M (GTG→ATG) | *xseA* → | exodeoxyribonuclease VII large subunit |
| Chromosome | 3,608,932 | T→C | 100.0% | D109G (GAC→GGC) | *gmhA* ← | phosphoheptose isomerase |
| Chromosome | 3,647,867 | C→T | 100.0% | A347V (GCT→GTT) | *pssA* → | phosphatidylserine synthase |
| Chromosome | 3,655,609 | T→C | 100.0% | K553E (AAA→GAA) | *clpB* ← | protein disaggregation chaperone |
| Chromosome | 3,739,401 | C→A | 100.0% | G70G (GGG→GGT) | *ydjJ* ← | Zinc‑binding dehydrogenase |
| Chromosome | 3,886,839 | T→C | 100.0% | E468E (GAA→GAG) | *ibeB* ← | outer membrane efflux lipoprotein |
| Chromosome | 3,979,205 | G→A | 100.0% | D130N (GAT→AAT) | *degQ* → | protease |
| Chromosome | 4,240,790 | A→T | 98.9% | I67I (ATT→ATA) | *YPO3778* ← | Sec‑independent protein translocase protein TatA |
| Chromosome | 4,268,351 | C→A | 100.0% | intergenic (+30/‑83) | *YPO3801* → / → *YPO3802* | hypothetical protein/hypothetical protein |
| Chromosome | 4,268,358 | C→A | 100.0% | intergenic (+37/‑76) | *YPO3801* → / → *YPO3802* | hypothetical protein/hypothetical protein |
| Chromosome | 4,293,962 | T→C | 100.0% | intergenic (‑101/‑524) | *glpA* ← / → *glpQ* | sn‑glycerol‑3‑phosphate dehydrogenase subunit A/glycerophosphodiester phosphodiesterase |
| Chromosome | 4,579,183 | A→G | 100.0% | S93G (AGC→GGC) | *fdhD* → | formate dehydrogenase accessory protein |
| Chromosome | 4,624,135 | C→G | 100.0% | P391R (CCC→CGC) | *trmE* → | tRNA modification GTPase TrmE |
| 1 Numbers in parentheses refer to distance upstream or downstream of nearest gene  2 Arrows refer to gene orientation | | | | | | |

| **Indels present in Yp2126 relative to YpCO92** | | | | | | |
| --- | --- | --- | --- | --- | --- | --- |
| **Location** | **Position** | **Mutation** | **Read Frequency** | **Annotation** | **Gene** | **Gene Product Description** |
| Chromosome | 17 | Δ1 bp | 100.0% | intergenic (–/+254) | – / ← *YPO0001* | –/flavodoxin |
| Chromosome | 104,236 | Δ7 bp | 97.7% | intergenic (‑323/+1403) | *tnp* ← / ← *YPO0096* | transposase for the IS1541 insertion element/transposase/IS protein |
| Chromosome | 126,994 | Δ8 bp | 97.8% | intergenic (+33/+138) | *metF* → / ← *YPO0118* | 5,10‑methylenetetrahydrofolate reductase/transposase for insertion sequence IS1661 |
| Chromosome | 472,503 | Δ6 bp | 97.1% | intergenic (‑156/+214) | *YPO0450* ← / ← *YPO0451* | hypothetical protein/cation‑transporting P‑type ATPase |
| Chromosome | 490,038 | Δ1 bp | 98.7% | intergenic (‑366/‑166) | *YPO0462* ← / → *talB* | hypothetical protein/transaldolase B |
| Chromosome | 581,519 | +TTCAA | 96.5% | intergenic (+299/‑30) | *leuO* → / → *YPO0536* | leucine transcriptional activator/hypothetical protein |
| Chromosome | 1,234,971 | +A | 100.0% | coding (220/312 nt) | *YPO1087* → | prophage protein |
| Chromosome | 1,580,109 | 18 bp x 2 | 90.2% | duplication | *YPO1397* ← | hypothetical protein |
| Chromosome | 1,811,400 | Δ10 bp | 99.6% | intergenic (+232/+36) | *YPO1588* → / ← *y1062* | hypothetical protein/transposase for the IS285 insertion element |
| Chromosome | 2,005,547 | Δ3 bp | 95.4% | coding (1025‑1027/1395 nt) | *yoaE* → | hypothetical protein |
| Chromosome | 2,037,934 | Δ7 bp | 94.6% | coding (285‑291/423 nt) | *flhE* → | flagellar protein FlhE precursor |
| Chromosome | 2,204,701 | 2 bp→TA | 100.0% | intergenic (+55/‑245) | *YPO1942* → / → *YPO1943* | hypothetical protein/hypothetical protein |
| Chromosome | 2,204,704 | 5 bp→5 bp | 100.0% | intergenic (+58/‑239) | *YPO1942* → / → *YPO1943* | hypothetical protein/hypothetical protein |
| Chromosome | 2,204,710 | 2 bp→TG | 100.0% | intergenic (+64/‑236) | *YPO1942* → / → *YPO1943* | hypothetical protein/hypothetical protein |
| Chromosome | 2,204,715 | 2 bp→AT | 100.0% | intergenic (+69/‑231) | *YPO1942* → / → *YPO1943* | hypothetical protein/hypothetical protein |
| Chromosome | 2,204,738 | Δ1 bp | 93.0% | intergenic (+92/‑209) | *YPO1942* → / → *YPO1943* | hypothetical protein/hypothetical protein |
| Chromosome | 2,236,267 | Δ1,957 bp | 100.0% | deletion | *y1093*–*YPO1969* | *y1093, YPO1969* |
| Chromosome | 2,552,609 | +G | 100.0% | intergenic (+47/+94) | *bioD* → / ← *YPO2270* | dithiobiotin synthetase/voltage‑gated ClC‑type chloride channel ClcB |
| Chromosome | 2,552,628 | Δ1 bp | 100.0% | intergenic (+66/+75) | *bioD* → / ← *YPO2270* | dithiobiotin synthetase/voltage‑gated ClC‑type chloride channel ClcB |
| Chromosome | 2,578,007 | 7 bp x 2 | 93.3% | duplication | *YPO2292* ← / → *ilvB* | lipoprotein/acetolactate synthase catalytic subunit |
| Chromosome | 2,771,484 | Δ12 bp | 96.8% | intergenic (+100/+472) | *YPO2470* → / ← *YPO2471* | hypothetical protein/hypothetical protein |
| Chromosome | 2,984,686 | 2 bp→AC | 100.0% | intergenic (+158/‑718) | *cspB* → / → *YPO2660* | cold shock protein/solute‑binding protein |
| Chromosome | 2,984,693 | 2 bp→AC | 100.0% | intergenic (+165/‑711) | *cspB* → / → *YPO2660* | cold shock protein/solute‑binding protein |
| Chromosome | 3,208,218 | Δ24 bp | 99.4% | deletion | *xseA* | exodeoxyribonuclease VII large subunit |
| Chromosome | 3,471,435 | Δ1 bp | 94.0% | intergenic (‑960/+138) | *ddhC* ← / ← *ddhA* | CDP‑4‑keto‑6‑deoxy‑D‑glucose‑3‑dehydratase/glucose‑1‑phosphate cytidylyltransferase |
| 1 Numbers in parentheses refer to distance upstream or downstream of nearest gene  2 Arrows refer to gene orientation | | | | | | |
| **Indels present in Yp2126 relative to YpCO92** | | | | | | |
| **Location** | **Position** | **Mutation** | **Read Frequency** | **Annotation** | **Gene** | **Gene Product Description** |
| Chromosome | 3,773,758 | Δ9 bp | 98.7% | coding (2373‑2381/2793 nt) | *barA* → | hybrid sensory histidine kinase BarA |
| Chromosome | 4,287,549 | Δ19 bp | 98.3% | intergenic (+8/+58) | *zntA* → / ← *YPO3821* | zinc/cadmium/mercury/lead‑transporting ATPase/sulfur transfer protein SirA |
| Chromosome | 4,296,703 | 8 bp x 2 | 91.9% | duplication | *YPO3828* ← | hypothetical protein |
| Chromosome | 4,487,107 | Δ6 bp | 97.1% | coding (1659‑1664/3213 nt) | *yapE* ← | autotransporter protein |
| Chromosome | 4,525,386 | Δ9 bp | 97.9% | intergenic (‑199/+322) | *yhjW* ← / ← *YPO4014* | phosphoethanolamine transferase/hypothetical protein |
| 1 Numbers in parentheses refer to distance upstream or downstream of nearest gene  2 Arrows refer to gene orientation | | | | | | |
